# Second-Trimester Pregnancy Loss at an Urban Hospital

**DOI:** 10.1080/10647440300025508

**Published:** 2003

**Authors:** Debra S. Heller, Charlene Moorehouse-Moore, Joan Skurnick, Rebecca N. Baergen

**Affiliations:** 1 Department of Pathology and Laboratory Medicine UMDNJ – New Jersey Medical School 185 South Orange Avenue PO Box 1709 Newark NJ 07101 USA; 2 Department of Obstetrics Gynecology and Women's Health Newark NJ USA; 3 Department of Preventive Medicine and Community Health UMDNJ New Jersey Medical School Newark NJ USA; 4 Department of Pathology and Laboratory Medicine Weill Medical College of Cornell University New York NY USA

## Abstract

*Objectives:* Second-trimester spontaneous pregnancy losses are less common than first-trimester losses, and are often associated with ascending infection and/or acute chorioamnionitis. A Medline search revealed only two large studies published in the recent literature, reporting incidences of chorioamnionitis of 39.3% and 58.2%, respectively. These studies did not address the use of histopathology for the identification of organisms. Since ascending infection is likely to be a significant cause of second-trimester loss in the inner-city population at the University Hospital in Newark, New Jersey, we sought to evaluate the usefulness of stains for microorganisms, which are rarely utilized on these specimens.

*Methods:*  Retrospective review of the medical records and pathologic material for cases of spontaneous abortions seen at the University Hospital in Newark between January 1999 and March 2001 was undertaken. Stains for microorganisms were performed on archival placental tissue for cases with histologic acute chorioamnionitis.

*Results:* A total of  67 cases were available for review, of which 38 cases (56.7%) showed histologic acute chorioamnionitis, similar to the rates in one previous study, but significantly higher than those in the other (*p* = 0.01). Of 25 cases with histological chorioamnionitis for which appropriate fetal material was available, 13 cases (52%) showed polymorphonuclear leukocytes (PMNs) in the fetal lungs, one case (4%) showed PMNs in the fetal stomach, and seven cases (28%) showed PMNs in both the lung and the stomach. Of the 38 cases with chorioamnionitis, Gram stains showed Gram-positive cocci in six cases, two of which were culture positive for group B streptococcus. Warthin–Starry stains showed filamentous organisms consistent with *Fusobacterium * sp. in the placenta in three cases.

*Conclusions:* Acute chorioamnionitis is associated with second-trimester pregnancy loss at this inner-city hospital, and may be related to the high incidence of risk factors in this population. A small proportion of cases can be further characterized by the inclusion of Gram and Warthin–Starry stains in the evaluation. Selection of cases with histologic acute chorioamnionitis for further study with special stains may provide additional information on the causative organism.


*Infect Dis Obstet Gynecol 2003;11:117–122*

### Second-trimester pregnancy loss at an urban hospital

**Debra S. Heller[1], Charlene Moorehouse-Moore[2], Joan Skurnick[3] and Rebecca N. Baergen[4]**

[1]*Department of Pathology and Laboratory Medicine,*
[2]*Department of Obstetrics, Gynecology and Women's Health,*
[3]*Department of Preventive Medicine and Community Health, UMDNJ New Jersey Medical School, Newark, NJ and*
[4]*Department of Pathology and Laboratory Medicine, Weill Medical College of Cornell University, New York, NY*

***Objectives:*** Second-trimester spontaneous pregnancy losses are less common than first-trimester losses, and are often associated with ascending infection and/or acute chorioamnionitis. A Medline search revealed only two large studies published in the recent literature, reporting incidences of chorioamnionitis of 39.3% and 58.2%, respectively. These studies did not address the use of histopathology for the identification of organisms. Since ascending infection is likely to be a significant cause of second-trimester loss in the inner-city population at the University Hospital in Newark, New Jersey, we sought to evaluate the usefulness of stains for microorganisms, which are rarely utilized on these specimens.
***Methods:*** Retrospective review of the medical records and pathologic material for cases of spontaneous abortions seen at the University Hospital in Newark between January 1999 and March 2001 was undertaken. Stains for microorganisms were performed on archival placental tissue for cases with histologic acute chorioamnionitis.
***Results:*** A total of 67 cases were available for review, of which 38 cases (56.7%) showed histologic acute chorioamnionitis, similar to the rates in one previous study, but significantly higher than those in the other ($p = 0.01$). Of 25 cases with histological chorioamnionitis for which appropriate fetal material was available, 13 cases (52%) showed polymorphonuclear leukocytes (PMNs) in the fetal lungs, one case (4%) showed PMNs in the fetal stomach, and seven cases (28%) showed PMNs in both the lung and the stomach. Of the 38 cases with chorioamnionitis, Gram stains showed Gram-positive cocci in six cases, two of which were culture positive for group B streptococcus. Warthin–Starry stains showed filamentous organisms consistent with *Fusobacterium* sp. in the placenta in three cases.
***Conclusions:*** Acute chorioamnionitis is associated with second-trimester pregnancy loss at this inner-city hospital, and may be related to the high incidence of risk factors in this population. A small proportion of cases can be further characterized by the inclusion of Gram and Warthin–Starry stains in the evaluation. Selection of cases with histologic acute chorioamnionitis for further study with special stains may provide additional information on the causative organism.

Key words: CHORIOAMNIONITIS; SPONTANEOUS ABORTION; SECOND TRIMESTER; PREGNANCY

---

Correspondence to: Debra Heller, MD, Department of Pathology-UH/E158, UMDNJ – New Jersey Medical School, 185 South Orange Avenue, PO Box 1709, Newark, NJ 07101, USA. Email: hellerds@umdnj.edu

© 2003 The Parthenon Publishing Group 

Up to 50% or more of all pregnancies will spontaneously abort, with the majority of these pregnancy losses occurring in the first trimester. First-trimester spontaneous abortions are often due to genetic or chromosomal anomalies. Spontaneous losses in the second trimester are less common and have been less well studied. However, it is known that many of them are associated with an ascending infection and/or acute chorioamnionitis. Although chorioamnionitis was well characterized in the older literature, a Medline search revealed only two large studies in the recent English-language literature evaluating clinical, histological and microbiological aspects of second-trimester pregnancy loss. Those studies reported rates of chorioamnionitis of 39.3% and 58.2%, respectively[1,2]. The University Hospital in Newark, New Jersey serves an inner-city population, and therefore ascending infection would probably be a common cause of second-trimester abortion in this population. In this study we sought to identify associated risk factors and evaluate the utility of histological stains for microorganisms, which are rarely used on these specimens.

### MATERIALS AND METHODS

A retrospective study was performed with Institutional Review Board approval. Second-trimester pregnancy losses between 13 and 21 weeks' gestation occurring between January 1999 and March 2001 at the University Hospital were identified from a computer search of pathology records. Second-trimester losses at less than 20 weeks are evaluated as surgical specimens at our institution, and all of them receive a gross and microscopic pathology examination. All cases were submitted as less than 20 weeks. However, a rare case was better classified as 21 weeks by measurements, in the absence of a reliable last menstrual period. A total of 67 medical records were reviewed, and fetal and maternal factors contributing to the loss were recorded, including race, maternal age, gravidity, previous pregnancy history, prenatal care, substance abuse, history of sexually transmitted diseases (chlamydia, syphilis, herpes, HIV, gonorrhea), presenting symptoms, and results of genital bacterial cultures. Slides of the placenta and of the fetus, where available, were reviewed on each for standardization by a single pathologist (DSH). Proportions are presented with 95% confidence intervals based on the binomial distribution. Fisher's exact test was used to compare the incidence of chorioamnionitis with rates in previously published studies. In addition, stains for microorganisms (Brown–Brenn, a Gram stain; Warthin–Starry, a silver stain that can be used to identify spirochetes) were performed on placental sections in cases with histologic chorioamnionitis as well as a sampling of negative controls, consisting of second-trimester placentas without chorioamnionitis. The Gomori methenamine silver stain (GMS) for fungal organisms was performed on a random subset of cases, as it was anticipated that the yield would be low.

### RESULTS

The demographics are summarized in Table 1. Cases of second-trimester spontaneous abortion who were treated at our hospital during the study period were the subjects of this study. The patients most commonly presented to the Emergency Room, and this was often the first time these patients were seen. Of a total of 77 requested charts, 67 charts were available for review. In total, 64 patients (95.5%) were African–American, corresponding to the population served by the hospital. The mean maternal age was 26.6 years

**Table 1** Demographics of the study population

| | |
|---|---|
| African–American | 95.5% |
| Mean maternal age | 26.6 |
| Mean gestational age | 16 5/7 |
| Mean gravidity | 5 |
| Prior preterm labor | 25% |
| One or more previous spontaneous abortions | 40% |
| One or more elective terminations of pregnancy | 48% |
| Obtained prenatal care | 54% |
| Illicit narcotic use | 34% |
| Smoking | 48% |
| Alcohol consumption | 12% |
| Prior history of chlamydia | 12% |
| Prior history of gonorrhea | 7.5% |
| Positive genital cultures at delivery | 21% |

(range 14–42 years). The mean gestational age at the time of abortion was 16 5/7 weeks (range 13 weeks 4 days to 21 weeks). The mean gravidity was 5 (range 1–12), 25% of cases had a history of previous preterm labor, 40% had a history of one or more previous spontaneous abortions, and 48% had had one or more previous elective terminations of pregnancy. Only 54% of patients had received prenatal care. Substance abuse was common, as 34% of patients had a history of illegal narcotic use. Smoking and alcohol abuse were also common, with an incidence of 40% ($n = 32$) and 12% ($n = 8$), respectively. In total, 12% ($n = 8$) had a history of chlamydia infection, 7.5% ($n = 5$) had a history of gonorrhea infection and 1.5% ($n = 1$) had a history of herpes infection. None of the patients had a history of syphilis, and there was only one confirmed case of HIV positivity. The most common presenting symptom was pain, and bleeding and vaginal discharge of fluid were also frequent. Culture results were available for 39 cases (58%). Overall, 21% ($n = 14$) of the patients had positive genital cultures at the time of delivery: 15% ($n = 10$) had positive cultures for group B streptococcus, 3% ($n = 2$) were positive for *Neisseria gonorrheae*, one patient (1.5%) had a positive culture for organisms consistent with bacterial vaginosis (*Gardnerella* sp. with mixed flora), and one patient (1.5%) had a culture that grew *Staphylococcus aureus*.

In total, 56.7% of the patients (38 cases) showed histologic acute chorioamnionitis (95% CI, 44.0–68.8%), and 26% of the patients with histologic acute chorioamnionitis had positive genital cultures. Of the 38 cases of histologic chorioamnionitis, 25 cases had histologically evaluable fetal lung and stomach. Of these cases, 52% ($n = 13$) showed polymorphonuclear leukocytes (PMNs) in fetal lungs, 4% ($n = 1$) showed PMNs in fetal stomach, and 28% ($n = 7$) showed PMNs in both lung and stomach (Figure 1). Gram and Warthin–Starry stains for microorganisms were performed on the 38 placentas with histologic chorioamnionitis as well as on nine randomly selected placentas without chorioamnionitis, which served as controls. The controls were all negative for microorganisms, although one was positive for group B streptococcus on genital culture. Gram stains showed Gram-positive cocci in 16% ($n = 6$) of the cases with acute chorioamnionitis, two of which were culture positive for group B beta streptococcus (Figure 2). Warthin–Starry stains showed filamentous organisms consistent with *Fusobacterium* sp. in 8% ($n = 3$) of cases, one of which was culture positive for group B streptococcus (Figure 3). GMS stain failed to identify any pathogenic organisms in the subset of cases stained ($n = 19$), and was discontinued due to lack of yield. A comparison of genital cultures and histochemical stains is shown in Table 2. Only four karyotypes were recorded, and two were associated with chromosomal abnormalities. In total, 33% ($n = 22$) of the patients had retained placenta that required intervention, and one patient required blood transfusion. Twelve patients were febrile, with temperatures of up to 102.8°F (39.33°C). However, as many of these patients received prostaglandin therapy, no interpretation of these findings was possible.

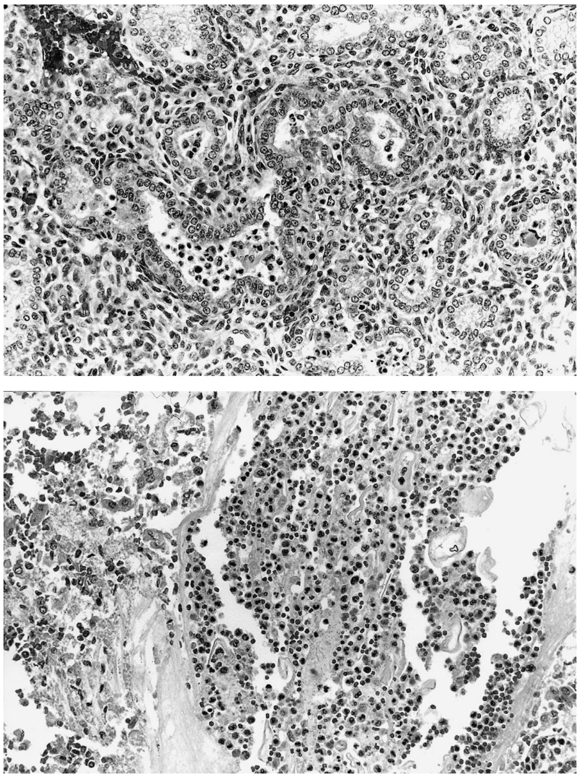

**Figure 1** Polymorphonuclear leukocytes (PMNs) were seen in (a) fetal lungs and (b) stomach in some cases

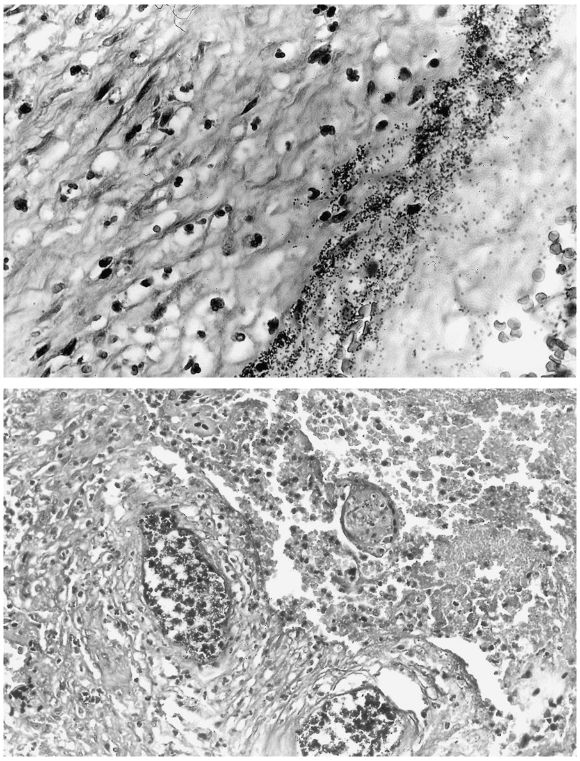

**Figure 2** (a) Gram stain of membranes in a case of culture-confirmed beta streptococci showing Gram-positive cocci (Gram stain); (b) bacteria seen in fetal villous vessels in the same case, consistent with fetal bacteremia (Gram stain)

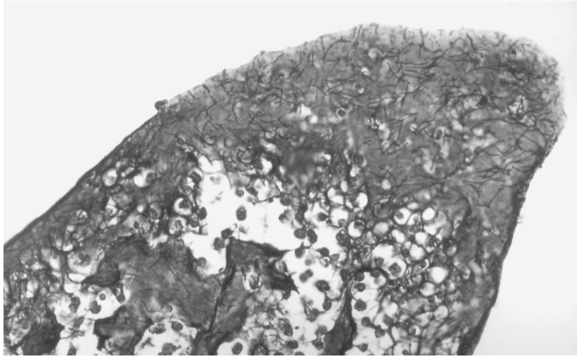

**Figure 3** Organisms consistent with *Fusobacterium* sp. seen in fetal membranes (Warthin–Starry stain)

### DISCUSSION

Histological chorioamnionitis is common in second-trimester pregnancy loss, and this is particularly true of the inner-city population of this study. In total, 56.7% (38 cases) showed histologic acute chorioamnionitis (95% CI, 44.0–68.8%), similar to the 58.2% rate (32 of 55 cases; 95% CI, 44.1–71.4%) found by Rudbeck and co-workers[2], but significantly higher than the 39.3% rate (166 of 422 cases; 95% CI, 34.7–44.2%) reported by Gaillard and co-workers[1] ($p = 0.01$). It should be noted that the study population of Rudbeck and co-workers[2] was drawn from a university hospital in Aarhus, Denmark, while the series of Gaillard and co-workers[1] was from a mix of urban and rural French women, and thus our population is probably more similar to that of Rudbeck and co-workers. Both series included second-trimester losses up to 27 weeks, which was somewhat later than our maximum age of 21 weeks. In Gaillard's series[1], as in ours, the most common single organism identified was group B streptococcus. However, mycoplasma was also a frequent isolate in their study. We found that performing special stains for microorganisms, which are often not undertaken in routine practice, was a high-yield procedure, confirming and in some cases providing the only information on the causative organism. This is particularly important in view of the suggestion by Rudbeck and co-workers[2] of possible lack of value of routine culturing. However, in that study, as in ours, cultures for mycoplasma and *Ureaplasma* sp. were not performed. We were interested in identifying cases of *Fusobacterium* sp., a less well-known cause of chorioamnionitis in term gestations, which was also shown by Altshuler and Hyde in 1985 to be associated with chorioamnionitis in prematurity[3]. Additional staining uncovered three cases of *Fusobacterium* sp. in our series, which were not previously identified by routine staining.

**Table 2** Comparison of histologic stains for microorganisms and genital culture results

| | *Positive culture* | *Negative culture* | *No culture performed* |
|---|---|---|---|
| Histochemical stain positive | 4 | 3 | 2 |
| Histochemical stain negative | 6 | 12 | 11 |

Our population is predominantly African–American, and these women bear a major burden of pregnancy loss. According to New Jersey State health statistics, infant mortality is three times greater in African–Americans than in Caucasians.

In 1998, the fetal mortality rate per 1000 (calculated for fetuses aged 20 weeks or older) for African–American women was 13%, compared with 5.6% for Caucasians. A risk factor for second-trimester loss is bacterial vaginosis, which is known to be more prevalent in African–Americans than in Caucasians[4]. We attempted to evaluate the role of bacterial vaginosis in our study by reviewing a recent pap smear for the presence of clue cells. However, as many of the patients were seen for the first time in our Emergency Room when they presented with a spontaneous abortion, insufficient information was available to allow us to draw conclusions. Other risk factors in this population are surrogate markers of exposure to infectious agents, including low socioeconomic status, substance abuse, and a history of other sexually transmitted diseases.

Acute chorioamnionitis is a major risk factor for preterm labor and preterm birth due to increased production of cytokines, prostaglandins and metalloproteinases[5]. Sebire and co-workers[6] postulated that localized inflammation of the decidua and chorion without amniotic involvement is actually the leading cause of second-trimester loss due to activation of cytokines merely by choriodecidual inflammation. Thus identification of acute chorioamnionitis and the causative organism is of clinical importance. Chorioamnionitis is often only present focally on histologic examination, and if the area of inflammation is not sampled, it may be missed. In these cases, it has been our experience that acute inflammatory cells in the fetal lungs may then be the only clue to diagnosis of an ascending infection. Of the 25 cases for which fetal tissue was available, we found that in 20 cases PMNs were present in fetal air spaces. It has been suggested that the presence of acute inflammatory cells in the fetal lungs is due to aspiration of inflammatory cells in the amniotic fluid, and thus does not represent a true fetal infection. However, Scott and co-workers[7] showed that 70–80% of PMNs in the air spaces of five male fetuses contained the Y-chromosome. It is generally considered that the presence of PMNs in the fetal lung is indicative of a fetal inflammatory response, and probably fetal infection. Although the fetus does not have the capacity to mount a full immunologic response to infection, it is interesting to note that the ability of the fetus to respond immunologically to infection is present to some degree prior to the age of viability. This is particularly important in view of the recent literature on the fetal inflammatory response and its association (through the release of cytokines and inflammatory mediators) with cerebral palsy in preterm infants[8].

Our series had a high percentage of patients with a history of spontaneous and elective abortions, and many patients had experienced recurrent reproductive losses. It has been shown that both prior induced abortions and, more importantly, prior spontaneous abortions are risk factors for a subsequent second-trimester loss[9]. Subclinical infection may persist, and this may lead to recurrent loss. Therefore identification of the organisms that are present at the first loss may be helpful in preventing future losses. Reinfection is another possibility, given the high incidence of sexually transmitted disease in this population.

However, etiologies for recurrent second-trimester loss are often different to those for initial second-trimester losses. Drakely and co-workers[10] studied a group of women in Liverpool with recurrent second-trimester loss. In 50% of cases no etiology was found, and 33% of cases were associated with antiphospholipid syndrome. Only 3% of patients were considered to have a possible infectious etiology, none of whom had chorioamnionitis at the time of the delivery. There were several limitations to this study. First, it was a retrospective review of records and pathologic material. Secondly, a full work-up was not performed on each case without acute chorioamnionitis.

In summary, our study has confirmed that acute chorioamnionitis is significantly associated with second-trimester pregnancy loss, and that it is particularly prevalent at this inner-city hospital. This may be due to the high incidence of risk factors in this population. Special stains for microorganisms, such as the Gram stain and the Warthin–Starry stain, can be helpful for ascertaining the causative organisms. However, GMS is probably not helpful in the evaluation of these cases. Tissue sections included both fetal and maternal surfaces. The positive staining of organisms corresponded to areas of inflammatory cells within the tissue, on the fetal surface, in either the membranes or the chorionic plate, and thus does not represent overgrowth of organisms, which would lack an inflammatory tissue response. Some of the cases with inflammation did not have organisms identified by the special stains, highlighting the value of performing cultures as well.

The question arises as to whether anything can be done to improve outcomes in this population. Sebire[6] stated that no screening methods are currently available to detect all cases of acute chorioamnionitis and choriodecidual inflammation. Although this may indeed be the case, certainly adequate prenatal care and healthy lifestyle choices are likely to reduce this problem significantly until the interaction of cytokines and other mediators with preterm labor and delivery is better understood. In addition to the psychological impact of pregnancy loss, the large number of patients requiring intervention for retained placenta constitutes a significant health risk.

### REFERENCES

1. Gaillard DA, Paradis P, Lalleman AV, *et al.* Spontaneous abortions during the second trimester of gestation. *Arch Pathol Lab Med* 1993;117:1022–6
2. Rudbeck RH, Henriques U. Fetal and perinatal infections: a consecutive study. *Pathol Res Pract* 1992;188:135–40
3. Altshuler G, Hyde S. Fusobacteria. An important cause of chorioamnionitis. *Arch Pathol Lab Med* 1985;109:739–43
4. Meis PJ, Goldenberg RL, Mercer BM, *et al.* Preterm predilection study: is socioeconomic status a risk factor for bacterial vaginosis in black or in white women? *Am J Perinatol* 2000;17:41–5
5. Goldenberg RL, Andrews WW, Hauth JC. Choriodecidual infection and preterm birth. *Nutr Rev* 2002;60:S19–25
6. Sebire NJ. Choriodecidual inflammatory syndrome (CoDIS) is a leading, and under-recognized, cause of early preterm delivery and second trimester miscarriage. *Med Hypotheses* 2001;56:497–500
7. Scott RJ, Peat D, Rhodes CA. Investigation of the fetal pulmonary inflammatory reaction in chorioamnionitis, using an *in-situ* Y-chromosome marker. *Pediatr Pathol* 1994;14:997–1003
8. Dammann O, Leviton A. Role of the fetus in perinatal infection and neonatal brain damage. *Curr Opin Pediatr* 2000;12:99–104
9. Puyenbroek JI, Stolte CA. The relationship between spontaneous and induced abortion and the occurrence of second-trimester abortion in subsequent pregnancies. *Eur J Obstet Gynecol Reprod Biol* 1983;14:299–309
10. Drakeley AJ, Quenby S, Farquharson RG. Mid-trimester loss – appraisal of a screening protocol. *Hum Reprod* 1998;13:1975–80

Received 11/18/02; Accepted 05/01/03

## References

[PDF_00466] Altshuler G., Hyde S. (1985). Fusobacteria. An important cause of chorioamnionitis.. Arch Pathol Lab Med.

[PDF_00485] Dammann O., Leviton A. (2000). Role of the fetus in perinatal infection and neonatal brain damage.. Curr Opin Pediatr.

[PDF_00493] Drakeley A. J., Quenby S., Farquharson R. G. (1998). Mid-trimester loss--appraisal of a screening protocol.. Hum Reprod.

[PDF_00460] Gaillard D. A., Paradis P., Lallemand A. V., Vernet V. M., Carquin J. S., Chippaux C. G., Visseaux-Coletto B. J. (1993). Spontaneous abortions during the second trimester of gestation.. Arch Pathol Lab Med.

[PDF_00473] Goldenberg Robert L., Andrews William W., Hauth John C. (2002). Choriodecidual infection and preterm birth.. Nutr Rev.

[PDF_00469] Meis P. J., Goldenberg R. L., Mercer B. M., Iams J. D., Moawad A. H., Miodovnik M., Menard M. K., Caritis S. N., Thurnau G. R., Dombrowski M. P. (2000). Preterm prediction study: is socioeconomic status a risk factor for bacterial vaginosis in Black or in White women?. Am J Perinatol.

[PDF_00488] Puyenbroek J. I., Stolte L. A. (1983). The relationship between spontaneous and induced abortion and the occurrence of second-trimester abortion in subsequent pregnancies.. Eur J Obstet Gynecol Reprod Biol.

[PDF_00463] Rudbeck Røge H., Henriques U. (1992). Fetal and perinatal infections. A consecutive study.. Pathol Res Pract.

[PDF_00481] Scott R. J., Peat D., Rhodes C. A. (1994). Investigation of the fetal pulmonary inflammatory reaction in chorioamnionitis, using an in situ Y chromosome marker.. Pediatr Pathol.

[PDF_00476] Sebire N. J. (2001). Choriodecidual inflammatory syndrome (CoDIS) is the leading, and under recognised, cause of early preterm delivery and second trimester miscarriage.. Med Hypotheses.

